# Simulation-Based Training in Aortic Aneurysm Surgery: Toward a New Educational Standard

**DOI:** 10.7759/cureus.89611

**Published:** 2025-08-08

**Authors:** Abubakar I. Sidik, Maxim L Khavandeev, Vladislav Dontsov, Dmitriy A Filimonov, Grigorii Esion, Malik K Al-Ariki, Md Limon Hossain, MST Asia Ajgar Bithi, Nelson Nicolas, Salehk Amro Zuhair Salah

**Affiliations:** 1 Cardiovascular Surgery, Peoples' Friendship University of Russia, Moscow, RUS; 2 Cardiothoracic Surgery, Gusak Institute of Emergency and Reconstructive Surgery, Donetsk, RUS; 3 Cardiothoracic Surgery, Moscow Regional Research and Clinical Institute, Moscow, RUS; 4 Experimental Surgery, Gusak Institute of Emergency and Reconstructive Surgery, Donetsk, RUS; 5 Cardiothoracic Surgery, A.A. Vishnevskiy Hospital, Moscow, RUS; 6 Surgery, Peoples' Friendship University of Russia, Moscow, RUS; 7 Cardiology, I.M. Sechenov First Moscow State Medical University, Moscow, RUS; 8 Cardiovascular Medicine, Peoples' Friendship University of Russia, Moscow, RUS; 9 Cardiovascular Medicine, Moscow Multidisciplinary Clinical Center Kommunarka, Moscow, RUS

**Keywords:** aortic aneurysm, endovascular aneurysm repair, robotic-assisted surgery, simulation-based training, surgical education, virtual reality

## Abstract

Simulation-based training is transforming the education of vascular surgeons in the management of aortic aneurysms (AAs), addressing limitations in traditional apprenticeship models amid declining open surgical volumes and increasing reliance on complex endovascular techniques. This review explores the current landscape of simulation technologies, including computational modeling, fluid-structure interaction, patient-specific 3D printing, artificial intelligence, and robotic platforms. These tools enable high-fidelity, anatomically accurate, and physiologically realistic training environments. Validated simulations enhance surgical decision-making, device deployment strategies, and complication management without risk to patients. Despite barriers such as cost, variability in implementation, and limited outcome data, simulation holds promise as an essential component of future vascular surgery curricula. By combining technical innovation with personalized, data-driven training, simulation is poised to redefine competency development and improve surgical safety and efficacy.

## Introduction and background

The complexity of aortic aneurysm (AA) repair demands a high level of surgical expertise, making structured training an essential component of vascular surgery education. Given the high-risk nature of AA procedures, ensuring that surgeons are well-prepared through simulation-based training and competency-focused education is critical in improving patient outcomes [1]. Traditional apprenticeship models are being gradually supplemented or replaced by structured learning pathways that incorporate simulated environments, virtual reality (VR), artificial intelligence (AI), and robotic-assisted surgery to refine surgical skills in a low-risk setting before live patient interactions [2].

Over the years, surgical education has shifted toward competency-based training, where emphasis is placed on objective skill assessment, structured learning modules, and standardized performance benchmarks [3]. The latest European Society for Vascular Surgery (ESVS) guidelines highlight the importance of simulation-based training, recommending its integration into vascular surgery programs. These initiatives aim to improve surgical competency, enhance patient safety, and reduce perioperative complications by providing trainees with adequate hands-on experience in a controlled environment [4].

This article explores the growing role of simulation-based training in vascular surgery, focusing on AA repair. It highlights the benefits of simulation in enhancing surgical skills, meeting annual caseload requirements, and integrating cutting-edge technologies like VR, AI, and robotic-assisted surgery. The article underscores the importance of structured training and how emerging innovations are reshaping the future of AA surgical education and practice.

## Review

The importance of surgical training in AA management

Competency Requirements for AA Surgery

The management of AA requires specialized surgical expertise, given the complexity of both open surgical repair (OSR) and endovascular aneurysm repair (EVAR). Vascular surgeons must develop proficiency in both techniques to ensure optimal patient outcomes. Training programs should, therefore, include structured educational frameworks, simulation-based learning, and competency assessments to prepare surgeons for both elective and emergency AA repair [4,5].

To maintain high standards of care, AA treatment centers should perform a minimum of 30 AA repairs per year, with at least 15 cases of OSR and 15 cases of EVAR. This volume threshold ensures that surgeons gain adequate hands-on experience and maintain their technical proficiency. Additionally, hospitals managing complex AAs (such as juxtarenal or thoracoabdominal aneurysms) should perform at least 20 combined OSR and fenestrated/branched EVAR procedures annually [4]. These volume-based benchmarks are supported by studies showing that higher surgical caseloads are associated with lower perioperative mortality rates and improved long-term patient outcomes [6,7].

Furthermore, structured fellowship training is essential for achieving proficiency in complex vascular procedures. Postgraduate fellowships should include dedicated training in advanced EVAR techniques, such as fenestrated and branched stent graft placement, as well as open surgical techniques for challenging cases. These fellowships should be conducted in high-volume centers with experienced faculty, providing trainees with comprehensive exposure to various aortic pathologies [8,9].

Challenges in Traditional AA Surgical Training

One of the major challenges in contemporary AA surgical training is the declining volume of open AA repairs, which has significantly reduced opportunities for hands-on learning. In the United States, for example, the number of OSRs declined by almost 80% from 2003 to 2013, largely due to the increasing adoption of EVAR [10]. By 2014, nearly half of senior vascular surgery trainees in the United States had performed fewer than five OSR procedures during their training [11]. This decline in operative exposure has raised concerns about the ability of future vascular surgeons to competently manage open surgical cases, particularly in ruptured AA (rAA) scenarios, where OSR remains a life-saving intervention [12].

Another critical issue is the learning curve associated with EVAR and complex endovascular procedures. Unlike OSR, which relies on direct anatomical exposure and surgical manipulation, EVAR requires expertise in fluoroscopic navigation, catheter-based interventions, and intraoperative imaging interpretation. This shift from open surgical techniques to wire-based endovascular skills presents a unique challenge for trainees, as these skills are not easily acquired through traditional surgical training methods [13].

Ethical considerations also pose challenges in AA training. Given the high-risk nature of AA procedures, there are strict limitations on the extent to which trainees can participate in real-life cases. The need to prioritize patient safety means that trainees often observe rather than actively perform key aspects of the surgery, limiting their hands-on experience. This has created an increasing demand for alternative training modalities, such as simulation-based education, to ensure safe and effective skill acquisition [12,14].

These challenges can be addressed by incorporating simulation-based training programs for both OSR and EVAR [4,15] and by the expansion of fellowship training programs and mandatory simulation training in vascular surgery curricula [4,5].

Simulation-based training: a game changer?

Integration of Simulation Into Surgical Education

The importance of integrating simulation into vascular surgery curricula, particularly as the volume of open AA repairs continues to decline, cannot be overemphasized; simulation-based training should be mandatory for vascular trainees, with a particular focus on EVAR, open aneurysm repair, and complex aortic procedures [4].

Many leading training institutions have already incorporated simulation into their certification programs, using structured assessments to ensure competency in key surgical techniques. Simulation-based exams, incorporating VR, 3D-printed models, and cadaveric simulations, are now being used as part of competency-based evaluations in many vascular surgery fellowships. A particularly promising development in simulation training is the integration of AI-driven learning platforms, which provide real-time feedback and personalized skill assessments [15,16].

Types of Simulation Models in AA Surgery

Surgical training simulators: A critical advancement in simulation training is the use of endovascular training simulators, which provide hands-on experience for EVAR procedures. These simulators allow trainees to practice wire and catheter manipulation, endograft deployment, and troubleshooting complications such as endoleaks. High-fidelity endovascular simulators have been shown to reduce radiation exposure, improve procedural planning, and enhance decision-making accuracy [17,18].

Among the latest innovations in hands-on endovascular training is the CathTrain™ simulator by SurgeonsLab (Bern, Switzerland), a portable endovascular flow simulator that replicates real hemodynamic conditions for training in catheter navigation and stent deployment. With transparent vascular pathways and pulsatile flow, it offers hands-on experience using real instruments [19]. Its realistic design makes it ideal for workshops and institutional use, bridging the gap between theory and live procedures in vascular surgery training.

**Figure 1:**
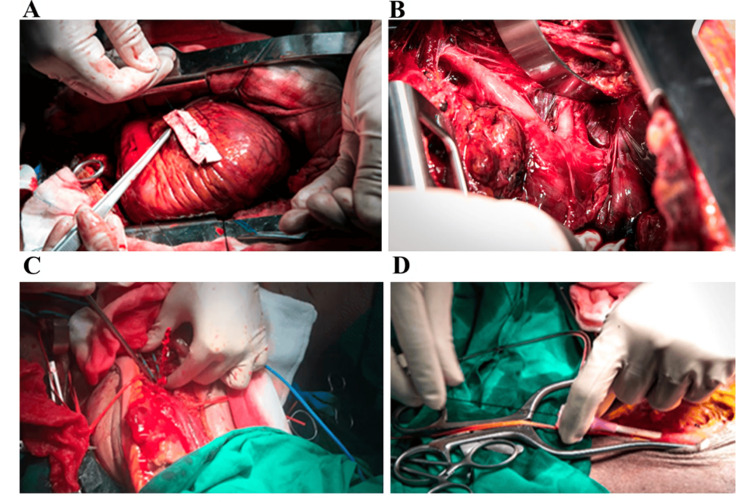
Cadaver-based simulation. (A) Demonstration of cardiac repair with felt strip reinforcement during manually simulated beating heart. (B) Exposure of arch branches via sternotomy. (C) Simulated vascular injury with pulsatile bleeding at the brachial artery. (D) Practicing REBOA with a pressure monitor. REBOA: resuscitative endovascular balloon occlusion of the aorta Reprinted under the terms of the Creative Commons Attribution 4.0 International License from Wannatoop et al. BMC Surg. 2022 [20].

Additionally, Cadaveric and Hybrid Simulation Labs serve as an important bridge between theoretical learning and real-life surgical application. Cadaver-based training provides an opportunity for tactile feedback and realistic anatomical dissection, crucial for mastering complex procedures such as vascular repair and damage control surgery. As illustrated in Figure 1, perfused cadaver models can simulate pulsatile bleeding, cardiac repair, and endovascular interventions like resuscitative endovascular balloon occlusion of the aorta (REBOA), offering unparalleled realism and high-stakes procedural practice in a controlled setting [20]. Hybrid simulation models, combining physical and digital interfaces, further enhance interactive procedural training by allowing real-time feedback and multi-modality integration [21].

**Figure 2:**
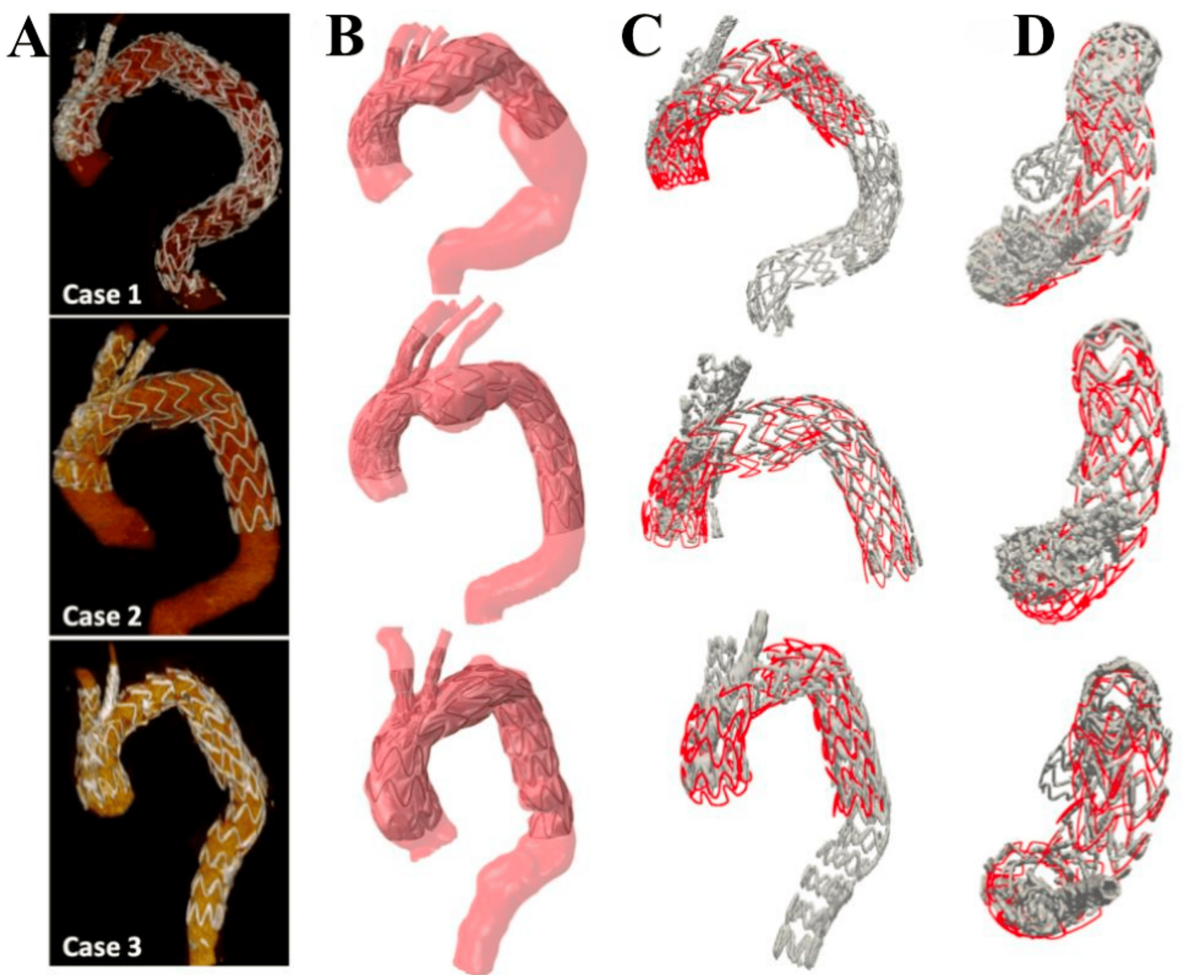
Comparison of simulation-based stent graft deployment with actual post-operative anatomy in three aortic arch cases. (A) 3D post-op CT reconstructions. (B) Simulation-predicted stent shapes. (C) Sagittal overlay of simulation (red) vs. actual CT (grey). (D) Transversal overlay views. Reprinted under the terms of the Creative Commons Attribution 4.0 International License from Derycke et al. J Clin Med. 2023 [22].

Numerical simulation models: Numerical simulation, including computational fluid dynamics (CFD) and finite element analysis (FEA), represents a growing frontier in AA surgical education. These simulations replicate patient-specific aortic geometries and blood flow dynamics using imaging data and mathematical modeling. By predicting areas of high wall stress or turbulent flow, numerical models inform procedural planning, endograft selection, and long-term risk assessment. While not hands-on in the traditional sense, these simulations enhance understanding of complex hemodynamics and device-aorta interactions [22]. As shown in Figure 2, patient-specific numerical simulation has demonstrated high accuracy in predicting the final deployed configuration of stent grafts, as validated by post-operative CT imaging.

Table 1 summarizes the key characteristics, advantages, limitations, and optimal applications of various simulation modalities currently employed in AA surgical education.

**Table 1:** Comparative overview of simulation models used in AA surgery training. AA: aortic aneurysm; CFD: computational fluid dynamics; EVAR: endovascular aneurysm repair; FEA: finite element analysis; FSI: fluid-structure interaction; OSR: open surgical repair; VR: virtual reality

Simulation Model	Key Features	Advantages	Limitations	Best Use Case
Endovascular Simulators	Hands-on catheter and wire navigation; real-time feedback	Improves technical skills; reduces radiation exposure	Limited to EVAR; expensive equipment	EVAR training for vascular trainees
Cadaveric and Hybrid Labs	Real anatomy with simulated bleeding and tactile feedback	High realism; excellent for trauma and open repair training	Ethical/logistical challenges; high maintenance cost	Advanced OSR and emergency procedure practice
Numerical Simulations (CFD/FEA/FSI)	Mathematical modeling of flow dynamics and wall stress	Enhances understanding of biomechanics and planning	Not hands-on; requires interpretation skills	Preoperative planning and academic instruction
Advanced Computational Modeling and AI	Patient-specific, AI-driven models with predictive analytics	Supports precision medicine and personalized rehearsal	Resource-intensive; limited validation for training use	AI-assisted, personalized training environments
VR	Immersive, interactive procedural scenarios	Adaptable to skill level; enables repeated practice	Lacks tactile feedback; moderate realism	Foundational training in both OSR and EVAR
3D-Printed Aneurysm Models	Physical replicas with pulsatile flow; imaging-compatible	Enhances spatial awareness; improves preoperative planning	Costly and time-consuming production	Patient-specific EVAR rehearsal and team training
Robotic Simulation Platforms	Joystick control of guidewires/catheters in robotic workflows	Improves precision; reduces operator fatigue and radiation	High cost; steep learning curve	High-tech institutions training for robotic EVAR

Advanced computational modeling and AI in AA simulation: Recent innovations in computational modeling have significantly broadened the scope of simulation-based training in AA surgery. What was once limited to procedural rehearsal has evolved into a multidisciplinary training platform incorporating hemodynamic analysis, biomechanical risk assessment, and patient-specific planning. Tools such as CFD, finite element modeling (FEM), and fluid-structure interaction (FSI) are increasingly used to simulate blood flow, aortic wall stress, and structural deformation within aneurysmal vessels. These simulations not only inform rupture risk prediction and device design but also enhance the educational value of surgical simulators by grounding them in biomechanical reality [23-26].

A central component of high-fidelity aneurysm simulation is the inclusion of physiologically accurate boundary conditions. Earlier models typically used generic inflow and outflow settings, which often failed to reflect actual patient physiology. Modern approaches now integrate patient-specific pressure waveforms, pulsatile flow data, and vascular resistance profiles to replicate the true hemodynamic environment [27,28]. This level of detail enhances both the clinical relevance of the simulation and the realism of the training experience, helping surgeons develop decision-making skills under lifelike conditions.

**Figure 3:**
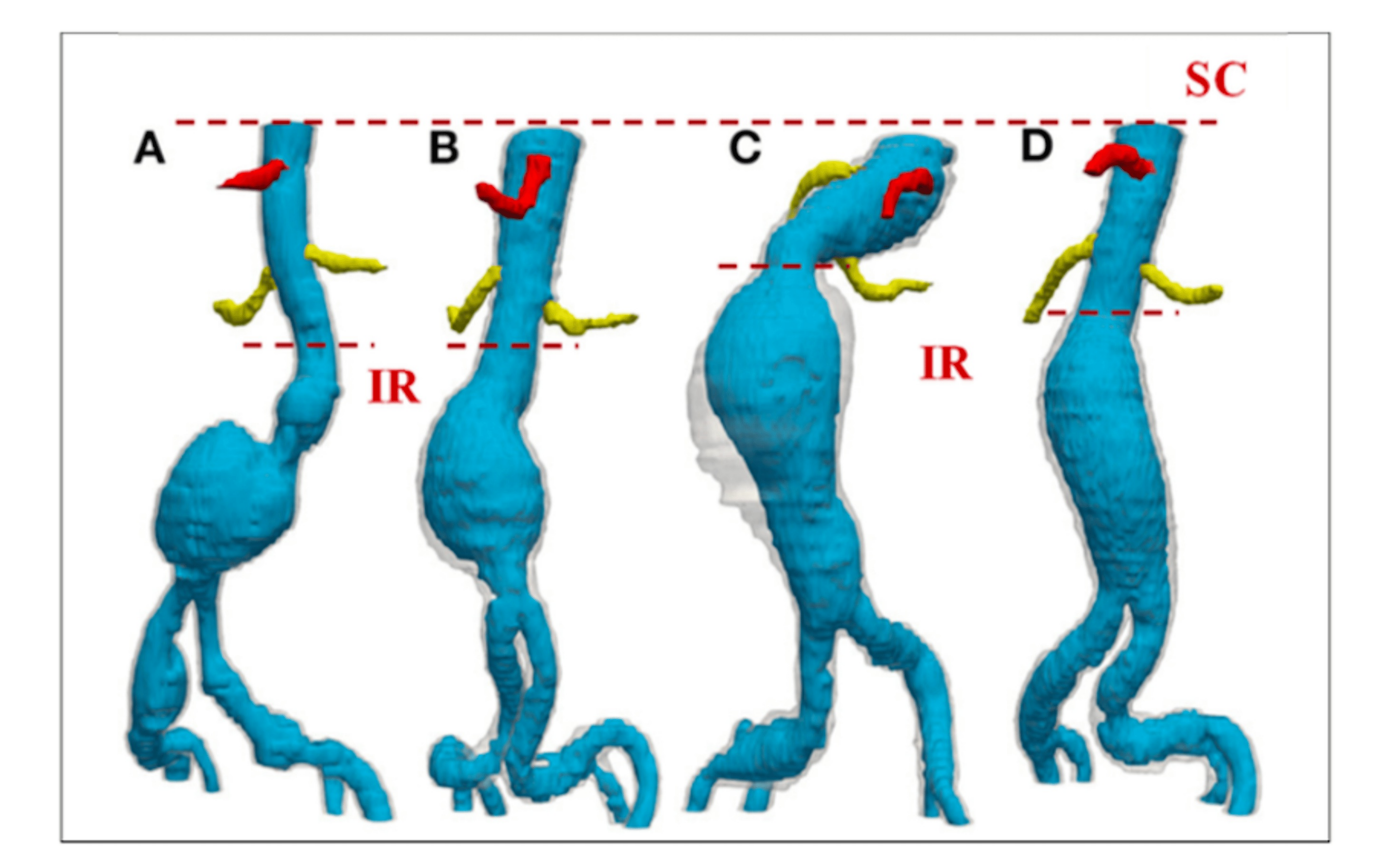
Patient-specific 3D aortic reconstructions generated using deep learning-based segmentation algorithms for four individuals (A-D). Dashed lines indicate the SC and IR anatomical landmarks relevant to aneurysm characterization and endograft planning. SC: superior; IR: infrarenal Reprinted under the terms of the Creative Commons Attribution 4.0 International License from Abdolmanafi et al. Front Cardiovasc Med. 2023 [29].

Another major advancement is the application of deep learning for automated image segmentation, which enables fast and precise construction of 3D patient-specific vascular models from medical imaging data. These reconstructions serve as the backbone of individualized simulation platforms. As illustrated in Figure 3, these models capture anatomical variations and highlight key surgical landmarks such as branch vessels, thrombus burden, and luminal geometry, providing learners with a realistic environment for preoperative planning and hands-on practice.

Machine learning (ML) is rapidly transforming the landscape of simulation-based training in AA surgery. Emerging studies show that ML algorithms can now automate anatomical segmentation, forecast aneurysm expansion, classify rupture risk, and even create fast surrogate models that replicate complex CFD outputs in real time [30]. These capabilities dramatically shorten the time from imaging to simulation, making it feasible to build personalized training scenarios and in silico rehearsal platforms tailored to individual patient anatomies.

The evolution of personalized aneurysm simulation is particularly impactful in surgical education, where combining imaging data with physiological signals and AI-based analytics enables the construction of virtual patients for high-fidelity training. These simulations replicate not only the geometric complexity of aneurysms but also their biomechanical behavior under surgical conditions, supporting personalized decision-making in graft sizing, access strategy, and risk anticipation [31]. As these platforms mature, simulation is positioned to become a central pillar of precision vascular surgery education and planning.

**Figure 4:**
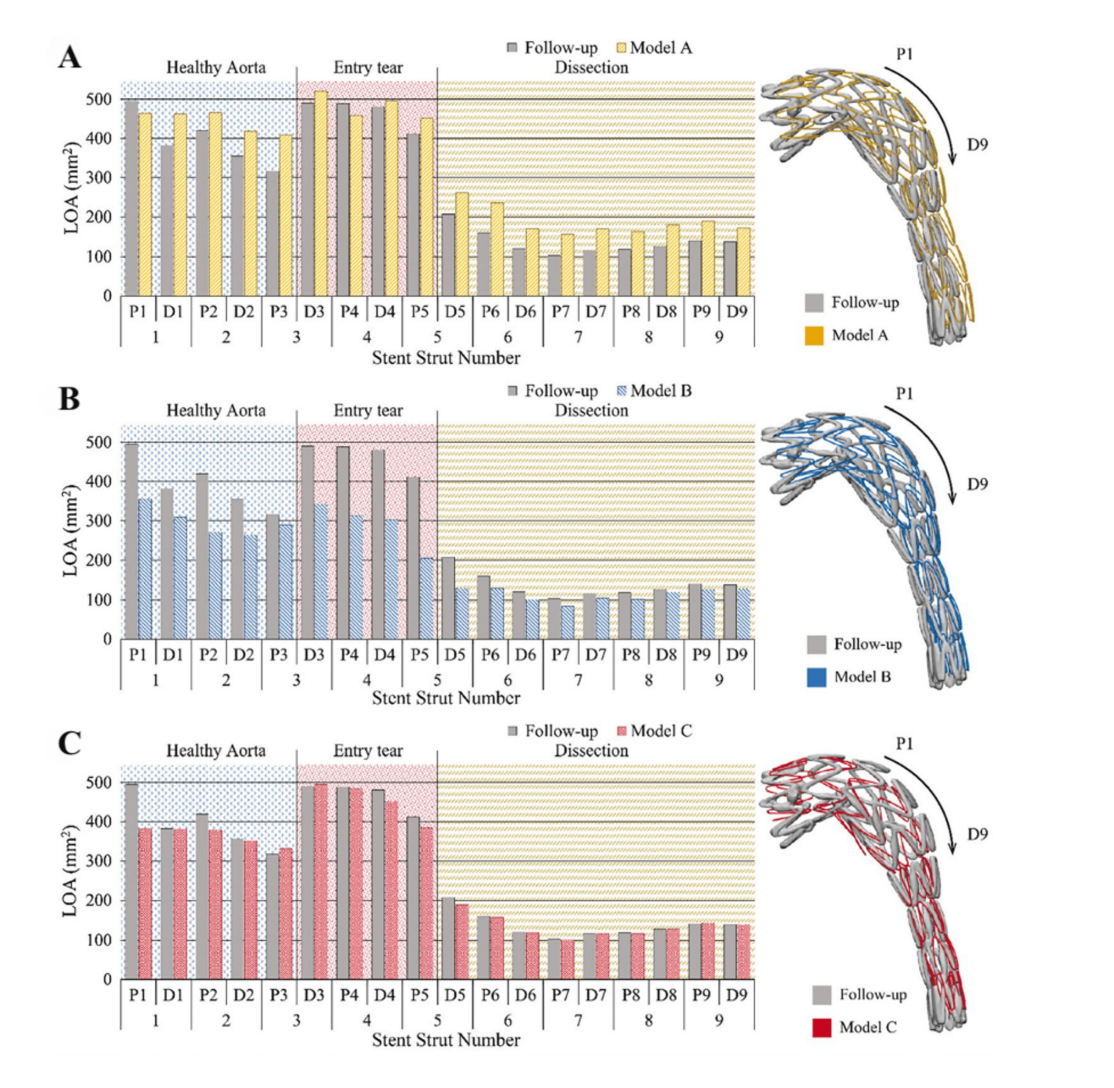
Validation of simulation models for TEVAR in TBAD. Comparisons of LOA at stent strut ends are shown for three models: (A) Model A (no pre-stress, no blood pressure), (B) Model B (pre-stress only), and (C) Model C (pre-stress + blood pressure). Model C demonstrated the closest agreement with follow-up CT data, emphasizing the importance of incorporating both biomechanical and hemodynamic factors. LOA: local open area; TEVAR: thoracic endovascular aortic repair; TBAD: type B aortic dissection Reprinted under the terms of the Creative Commons Attribution 4.0 International License from Kan et al. Biomech Model Mechanobiol. 2021 [32].

A critical aspect of these individualized simulations is the accurate modeling of biomechanical loading and device-artery interactions. In a landmark study, Kan et al. evaluated three finite element models of thoracic endovascular aortic repair (TEVAR) in TBAD, each incorporating different physiological assumptions. Only the model that included both aortic pre-stress and internal stent-graft pressure (Model C) reproduced the post-procedural configuration seen on follow-up CT scans with high fidelity (Figure 4) [32]. This underscores the need to embed biomechanical realism in simulation-based training environments, particularly when preparing trainees for complex endovascular repairs [32].

Accurate biomechanical representation is critical to the success of simulation-based training in AA surgery. Ramella et al. have shown that excluding arterial pre-stress from patient-specific finite element models results in severe underestimation of mechanical forces, specifically, aortic wall stress and strain were underestimated by over 150% in TEVAR and 160% in TAVI procedures [33]. Such deviations undermine the realism of simulated outcomes and could lead to misleading insights during both clinical planning and surgical training.

**Figure 5:**
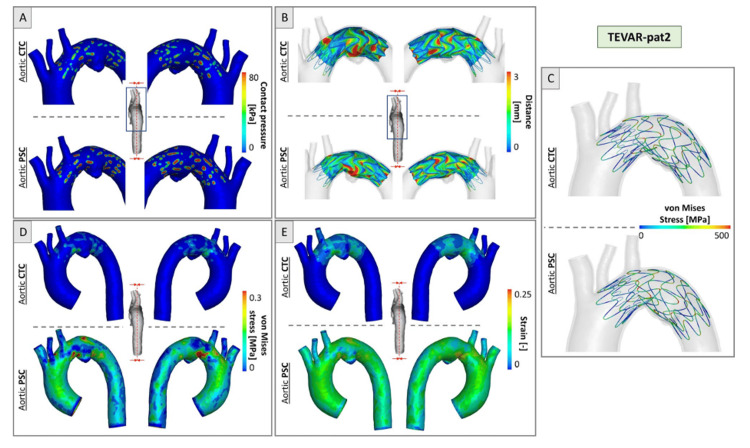
Simulation results for TEVAR-patient 2 comparing CTC and PSC configurations. (A) Contact pressure distribution between stent struts and the aortic wall. (B) Distance between the device and the aortic wall. (C) Von Mises stress distribution in stent struts. (D) Von Mises stress in the aortic wall. (E) Strain distribution in the aortic wall. TEVAR: thoracic endovascular aortic repair; CTC: computed tomography configuration (without arterial pre-stress); PSC: pre-stressed configuration (with arterial pre-stress) Reprinted under the terms of the Creative Commons Attribution 4.0 International License from Ramella et al. Biomech Model Mechanobiol. 2023 [33].

As visualized in Figure 5, the comparison between simulations with and without pre-stress, termed the CTC (non-prestressed) and PSC (prestressed) models, reveals significant differences in contact pressure, stent-aorta interaction, and tissue strain [33]. Ramella et al.’s work reinforces the notion that biomechanical preconditioning is not optional but essential for high-fidelity simulation. Incorporating arterial pre-stress into training platforms ensures that surgical trainees experience and respond to realistic feedback conditions, particularly during device deployment, risk assessment, and complication anticipation.

High-fidelity hemodynamic simulation is a cornerstone of effective simulation-based training in AA and dissection surgery. Zimmermann et al. developed a robust FSI model of type B aortic dissection (TBAD), validated through both in vitro perfused models and 4D-flow MRI [34]. Their study explored how manipulating entry and exit tear sizes affected hemodynamics: larger entry tears led to elevated false lumen pressure, wall shear stress, and flap stress, while larger exit tears facilitated hemodynamic decompression and laminar flow restoration. These nuanced biomechanical effects are essential for training surgeons to anticipate complex intraluminal dynamics and make informed decisions during TEVAR planning.

Critically, Zimmermann et al. validated their FSI simulations against real-world experimental measurements of inlet pressure and flow distribution across the aortic branches. As shown in Table 2, the residual errors between simulated and measured values were remarkably low, under 4.6% for pressure and 7.4% for flow splits, underscoring the reliability of this patient-specific modeling approach [34]. These validated metrics provide a strong foundation for using such simulations not only in research and planning but also in educational settings where realism and feedback accuracy are paramount.

**Table 2:** Hemodynamic validation results. Inlet pressure (mmHg) and flow splits (% of total outflow) for the three models. The FSI simulations matched target pressure values, systolic (P_sys_), diastolic (P_dias_), and mean (P^MAP^), with a relative error of ≤4.58% and an absolute error of ≤6.5 mmHg. Flow split ratios between outlets were matched in the FSI simulations with a relative error of ≤7.43% and an absolute error of ≤1.5%. FSI: fluid-structure interaction; BCT: brachiocephalic trunk; LCC: left common carotid; LSA: left subclavian artery; TBAD: type B aortic dissection; OR: original reconstruction; EN: endograft simulation; EX: extended endograft simulation Reprinted under the terms of the Creative Commons Attribution 4.0 International License from Zimmermann et al. Sci Rep. 2023 [34].

Model	Pressure Tuning at Inlet			Flow Split Tuning Across Outlets			
	P_sys _(mmHg)	P_dias_ (mmHg)	P_MAP _(mmHg)	BCT (%)	LCC (%)	LSA (%)	Outlet (%)
TBAD_OR_							
Simulated	127.5	59.1	86.1	15.5	3.2	5.6	75.7
Measured	126.4	60.0	86.1	15.0	3.1	5.4	76.5
Residual Error (%)	0.82	1.42	0.08	3.37	3.22	3.08	1.02
TBAD_EN_							
Simulated	141.9	72.7	95.7	12.3	3.0	6.1	78.6
Measured	137.7	69.9	96.2	11.4	2.8	5.7	80.1
Residual Error (%)	3.02	4.11	0.57	7.43	7.21	7.00	1.84
TBAD_EX_							
Simulated	148.6	75.1	100.4	11.7	3.3	5.0	80.1
Measured	142.1	73.5	98.9	10.9	3.1	4.6	81.4
Residual Error (%)	4.58	2.24	1.50	7.26	7.06	6.93	1.63

**Figure 6:**
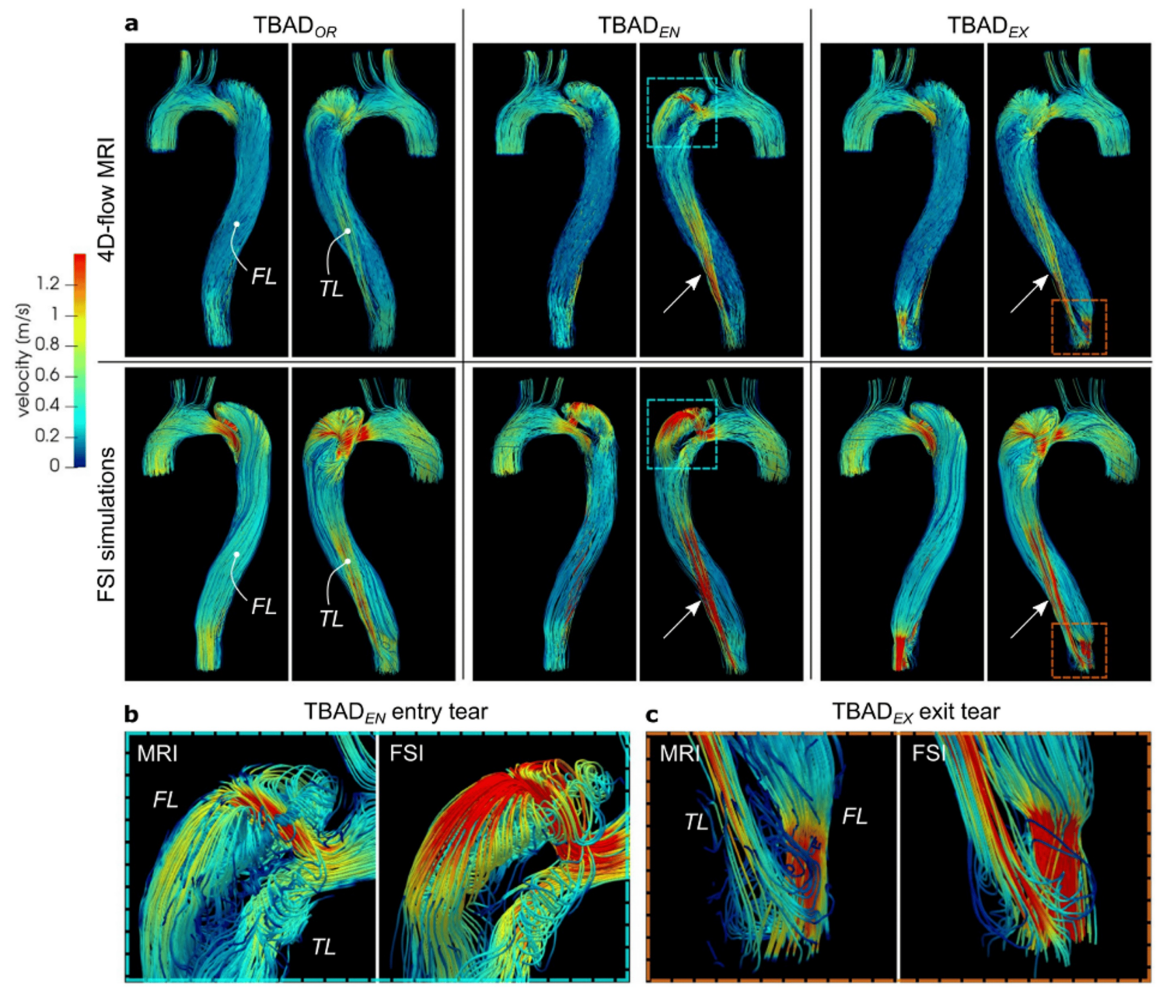
Streamline maps of hemodynamic effects. (A) Streamlines at peak systole (t=200 ms) rendered from 4D-flow MRI (top) and FSI-simulated (bottom) data. Each model exhibits unique local flow characteristics that show agreement between techniques. Key observations include: (i) increased flow velocities through the entry tear region, particularly in TBADEN, and local helical flow in the proximal TL and FL near the entry tear (blue box; close-up in (B)); (ii) increased TL flow velocity for the modified TBADEN and TBADEX models (arrows); (iii) flow jet through the small exit tear in TBADEX, with recirculating TL flow distal to the exit tear (orange box; close-up in (C)). FSI: fluid-structure interaction; TBAD: type B aortic dissection; OR: original reconstruction; EN: endograft simulation; EX: extended endograft simulation; TL: true lumen; FL: false lumen Reprinted under the terms of the Creative Commons Attribution 4.0 International License from Zimmermann et al. Sci Rep. 2023 [34].

These hemodynamic effects are further visualized in streamline maps generated from 4D-flow MRI and FSI simulations, which demonstrate distinct flow patterns and strong agreement between techniques across all tear configurations (Figure 6) [34].

**Figure 7:**
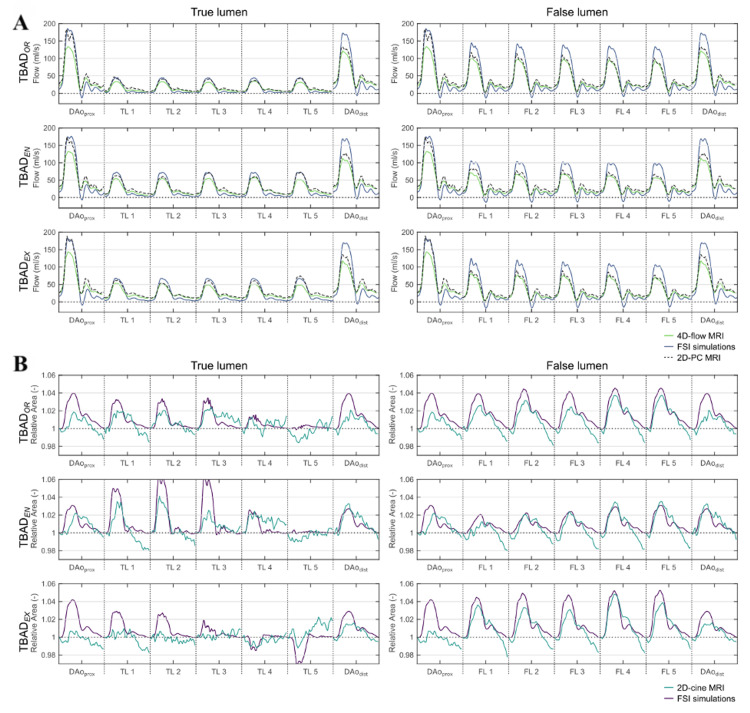
Time-resolved comparison of aortic flow and deformation between FSI simulations and experimental measurements for TBADOR, TBADEN, and TBADEX. (A) Flow rate waveforms in the true and false lumens as measured by 4D-flow MRI (green), FSI simulation (blue), and 2D-PC MRI (black dashed). (B) Relative cross-sectional area changes over the cardiac cycle measured using 2D-cine MRI (light blue) and predicted by FSI simulations (purple). The high degree of concordance across all metrics reinforces the validity of the FSI models for patient-specific simulation of aortic dissection. FSI: fluid-structure interaction; TBAD: type B aortic dissection; OR: original reconstruction; EN: endograft simulation; EX: extended endograft simulation Reprinted under the terms of the Creative Commons Attribution 4.0 International License from Zimmermann et al. Sci Rep. 2023 [34].

To further establish the fidelity of their patient-specific simulation, Zimmermann et al. compared time-resolved flow waveforms and dynamic vessel deformation between FSI predictions and multiple MRI modalities, including 4D-flow, 2D-PC, and cine MRI, across three dissection tear configurations. As shown in Figure 7, the strong agreement across all methods and time points reinforces the reliability of this simulation framework for training applications that demand both anatomical and hemodynamic accuracy [34].

VR in AA surgical training: VR has emerged as a powerful training tool in vascular surgery, offering highly immersive and interactive environments in which trainees can practice both open and endovascular AA repair. By simulating real-life surgical scenarios, VR enables trainees to visualize complex aortic anatomy, develop technical proficiency, and confront intraoperative challenges in a safe and controlled setting. This technology not only enhances anatomical understanding but also promotes the acquisition of fine motor skills essential for successful surgical outcomes [35].

One of the key advantages of VR training is its ability to provide real-time feedback on surgical techniques. This allows trainees to identify and correct errors promptly, refining their approach with each session. Additionally, VR supports repetitive practice without any risk to patients, thereby helping learners build muscle memory and surgical precision. The training modules can be tailored to different levels of difficulty, which makes them adaptable to individual learning curves and ensures continued progression as the trainee’s skills improve [36].

Several advanced VR platforms are currently being integrated into vascular surgery training curricula. Among them, the Simbionix ANGIO Mentor stands out for its realistic simulation of catheter-based interventions [37], while the VIST Endovascular Simulator offers detailed modules for EVAR planning and execution [38]. The Mentice VIST G7 is a high-fidelity endovascular simulator that supports the use of real clinical devices and provides advanced haptic feedback, further enhancing the realism of training environments. The growing adoption of these platforms in residency programs is helping to bridge the gap between theoretical knowledge and hands-on experience, ensuring that surgical trainees are adequately prepared for the high demands and risks associated with AA repair.

3D-printed aneurysm models: 3D aortic models have also emerged as a valuable tool for patient-specific preoperative planning. These models allow surgeons to practice complex AA repairs on anatomically accurate, patient-matched aortic replicas, improving operative decision-making and endograft selection. Research indicates that patient-specific rehearsal using 3D-printed models before EVAR leads to reduced procedural errors, improved graft positioning, and enhanced operative efficiency [39].

While initially developed for neurovascular applications, Biomodex’s EVA model technology is expanding into aortic simulation and offers immense value in AA surgical training. Its AI-based design converts imaging data into patient-specific vascular replicas with embedded synthetic blood flow systems and haptic feedback. This environment allows trainees to rehearse EVAR procedures using real surgical tools in a life-like setup, improving decision-making under simulated fluoroscopic guidance [40].

Recent advances in 3D printing have enabled the development of patient-specific aneurysm phantoms that accurately replicate complex vascular anatomy and support real-time hemodynamic simulation. When integrated into pulsatile flow loops, these models simulate near-physiological conditions, including arterial pressure, flow velocity, and compliance, providing a dynamic environment for investigating device behavior, flap mechanics, and procedural outcomes. These platforms are compatible with fluoroscopy, ultrasound, and CT imaging, allowing clinicians to rehearse TEVAR procedures using real delivery systems in a hybrid setting.

**Figure 8:**
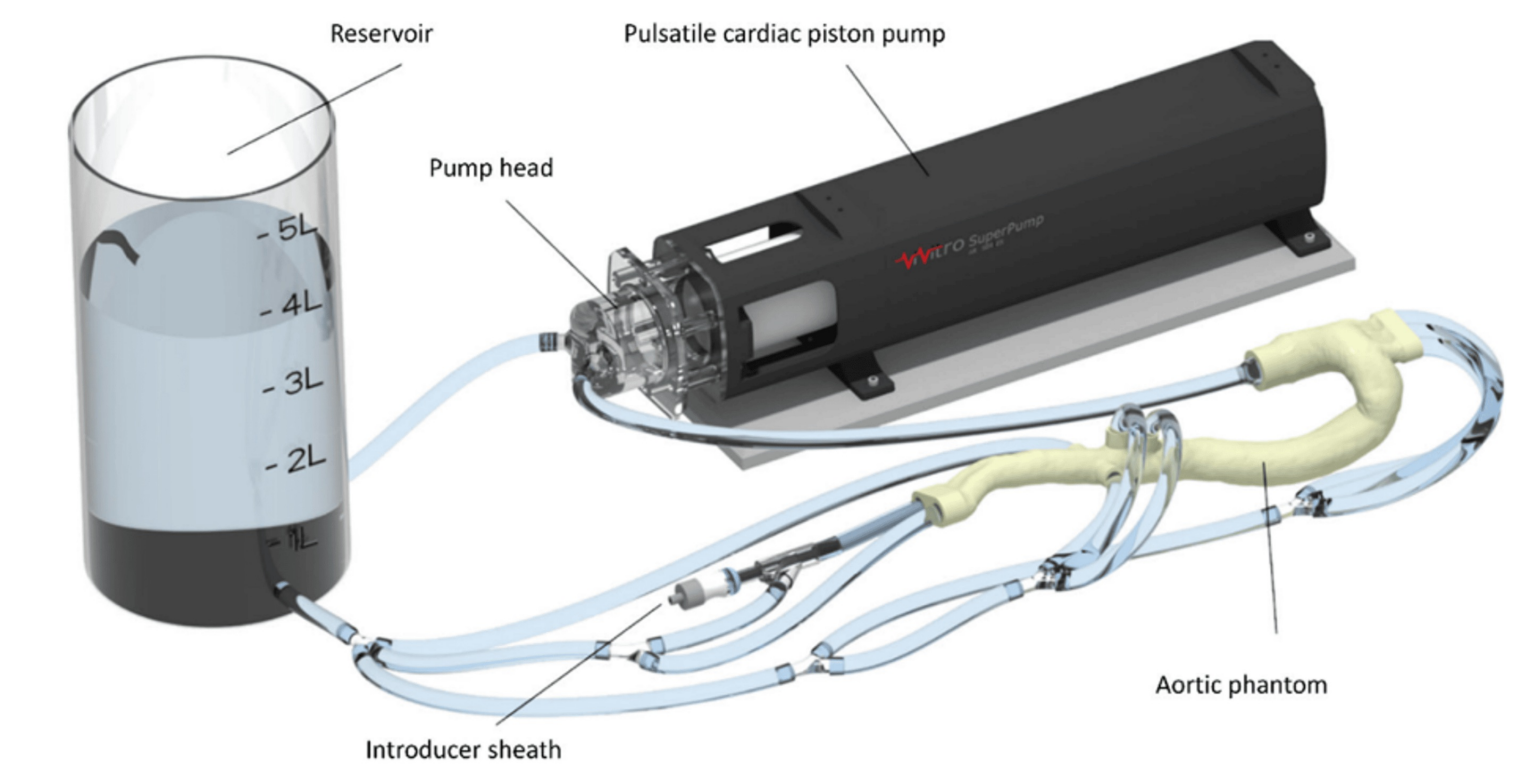
Aortic flow loop integrated with a patient-specific, 3D-printed aortic dissection phantom. A pulsatile cardiac piston pump drives hemodynamic flow through the phantom, simulating realistic vascular conditions. Each branching vessel is independently connected to a fluid reservoir, enabling pressure and flow measurements. The introducer sheath in the right iliac artery allows for catheter and stent-graft delivery during simulated TEVAR procedures. TEVAR: thoracic endovascular aortic repair Reprinted under the terms of the Creative Commons Attribution 4.0 International License from Mohl et al. Int J CARS. 2024 [41].

Such simulation environments offer substantial value in surgical education, device testing, and preoperative planning, particularly when teaching complex endovascular techniques. As shown in Figure 8, Mohl et al. developed a fully perfused, patient-specific model of TBAD, connected to a programmable cardiac pump and imaging, compatible for use in hybrid operating rooms. This approach brings high-fidelity, patient-specific training one step closer to clinical reality, bridging the gap between virtual modeling and hands-on procedural rehearsal [41].

AI-driven surgical planning and personalized training models: AI is playing a transformative role in surgical education by providing personalized training models that adapt to the skill level and learning pace of individual trainees. AI-assisted simulation platforms collect and analyze performance data to identify specific strengths and weaknesses in a trainee’s technique. Based on this analysis, the platforms can adjust difficulty levels and modify training scenarios accordingly. This adaptive learning approach allows for more efficient, targeted skill development and ensures that each trainee receives instruction tailored to their unique progress and needs [42,43].

Viz.ai contributes to surgical training indirectly by integrating AI-based AA detection into radiology workflows. Trainees learn how AI enhances diagnostic accuracy and speeds up clinical decision-making in time-sensitive cases like ruptured aneurysms. While not a simulation tool, it serves as an educational model for understanding how AI assists in early aneurysm identification and clinical triage. Exposure to this technology helps future vascular surgeons appreciate the broader impact of AI in care coordination, from image analysis to operative readiness [44,45].

ZedView® and Zed3D, powered by LEXI, offer hands-on surgical simulation for AA management using AI-enhanced anatomical reconstruction. Trainees use these tools to virtually navigate complex aneurysms, simulate stent-graft deployment, and assess anatomical suitability for EVAR. This AI tool plays a key role by automating vessel segmentation and identifying calcifications and landing zones, tasks that would otherwise be time-consuming and prone to human error [46].

Robotic assistance in AA surgery: Robotic-assisted surgery is increasingly gaining traction in vascular surgery, particularly for complex endovascular interventions. These robotic platforms significantly enhance surgical precision by offering improved control, greater instrument stability, and refined fine motor movements. In the context of AA repair, such enhancements help reduce the risk of intraoperative complications and contribute to better surgical outcomes [47].

One of the key advantages of robotic assistance lies in its capacity for superior endovascular navigation. This capability allows for highly accurate catheter and stent deployment, even in anatomically challenging cases. Additionally, robotic systems facilitate minimally invasive approaches, which can lead to reduced procedure times, lower radiation exposure for both patients and surgical teams, and fewer post-operative complications. In open AA repairs, robotics also offers improved dexterity and stability, critical features for performing delicate suturing and precise graft placement [40].

**Figure 9:**
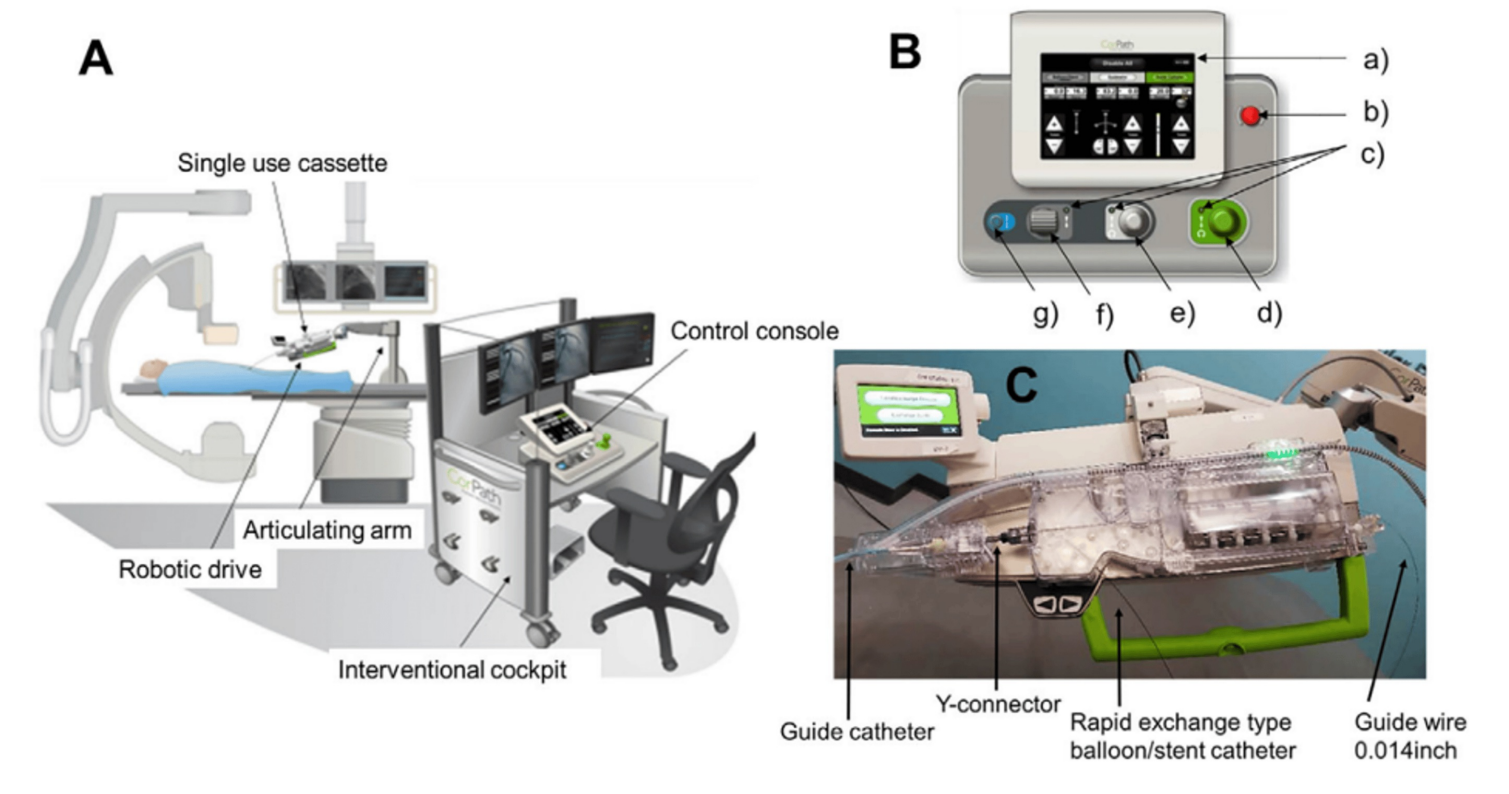
The CorPath GRX system. (A) Overview of the CorPath GRX system in the catheterization laboratory. (B) Control console. a) Touch screen, b) Emergency stop button, c) Active joystick LED, d) Guide catheter joystick, e) Guidewire joystick, f) Balloon/stent catheter joystick, and g) Turbo button. Reprinted under the terms of the Creative Commons Attribution 4.0 International License from Kagiyama et al. Intern Med. 2019 [48].

Advanced robotic platforms, such as the CorPath GRX system (Figure 9), are now being introduced into vascular surgery training programs. These systems provide trainees with opportunities to practice robotic controls and techniques in simulated environments, equipping them with hands-on experience before transitioning to live patient procedures [48,49].

The R-One robotic platform by Robocath, originally designed for coronary interventions, is now being evaluated for peripheral and aortic applications. As a training platform, R-One allows vascular trainees to experience joystick-based control of guidewires and balloon catheters, giving them a taste of what robot-assisted EVAR workflows might look like in the near future. It fosters an understanding of how robotic control can reduce operator fatigue, minimize radiation exposure, and provide precision in catheter-based AA interventions, especially during iliac access or stent limb placement [50].

Despite these promising developments, the broader integration of robotics into AA surgery is still limited. Barriers such as high equipment and maintenance costs, the steep learning curve associated with mastering robotic systems, and the need for further clinical validation currently hinder widespread adoption. Nonetheless, as technology continues to evolve and more data support its effectiveness, robotic-assisted surgery holds considerable promise for the future of vascular surgical care [47].

Advantages of Simulation in AA Training

Simulation-based education has been widely recognized for its role in enhancing technical proficiency and improving surgical decision-making, particularly in high-risk procedures like AA repair. One of the key benefits of simulation training is that it provides a risk-free environment for practicing complex interventions, enabling trainees to develop their skills without compromising patient safety [38].

Moreover, studies have demonstrated that simulation-based training significantly reduces procedural errors, particularly in EVAR. A randomized trial comparing conventional training with simulation-based EVAR training found that trainees who underwent simulation-based education had fewer errors in endograft deployment and improved procedural efficiency. Furthermore, simulation training has been associated with shorter fluoroscopy times, leading to reduced radiation exposure for both surgeons and patients [17,18].

Another major advantage of simulation is its role in standardizing vascular surgical education, ensuring that trainees across different institutions attain a consistent level of competency. The structured, reproducible nature of simulation-based learning allows for objective assessment of technical skills, ensuring that trainees meet the necessary proficiency benchmarks before performing procedures on patients [13].

Unresolved questions and future directions 

The incorporation of simulation-based training into vascular surgery is transforming the way AA repair is taught and practiced. However, challenges remain in standardizing curricula, improving accessibility, and evaluating long-term effectiveness. As surgical education continues to evolve, addressing these unresolved questions will be crucial for ensuring that simulation-based learning translates into improved patient outcomes [2,36].

Standardizing Simulation-Based Training

One of the major challenges facing modern vascular surgery training is the absence of a universally accepted simulation curriculum. Although many institutions have begun incorporating advanced tools such as VR, AI-assisted planning, and robotic training into their educational programs, there remains considerable variation in how these technologies are applied. This disparity is evident across different institutions, regions, and countries, making it difficult to ensure a consistent and uniform level of surgical competency among vascular surgeons globally [15,51].

A standardized simulation-based training framework could help address this issue by clearly defining the core competencies required for AA surgical training. Such a framework would also offer structured guidelines on how to effectively integrate VR, AI, and robotics into learning modules, ensuring that these innovations enhance rather than complicate the educational experience. Moreover, standardized simulation curricula would support global accreditation and benchmarking efforts, helping to ensure that trainees, regardless of geographic location, receive equivalent opportunities for skill development and assessment [13,17].

However, implementing these advanced technologies within existing training structures presents a number of logistical and financial hurdles. Many residency programs operate under constrained budgets and limited infrastructure, making it difficult to acquire and maintain high-fidelity simulation equipment. Without additional funding and institutional support, widespread integration of these technologies remains a challenge. Therefore, the development of cost-effective training solutions that strike a balance between technological sophistication and practical feasibility will be essential to achieving broader adoption and, ultimately, improving the quality and consistency of vascular surgical education worldwide [15,38].

Cost Versus Accessibility of Advanced Training Technologies

While VR, AI-based decision support systems, and robotic surgical platforms offer transformative training opportunities in vascular surgery, their high cost remains a significant barrier to widespread adoption. High-fidelity simulators, particularly endovascular VR platforms and robotic training systems, often require substantial financial investments. This limits their availability to large, well-funded academic institutions, leaving many smaller or resource-constrained programs without access to these valuable tools. To overcome this challenge and make advanced surgical training more accessible, several strategic solutions can be implemented [52,53]. These strategies are summarized in Table 3.

**Table 3:** Strategic solutions for widespread adoption of advanced training technologies.

Strategy	Description
Reduce Platform Costs	Encourage the development and adoption of open-source simulation software and more affordable hardware options to drive down the overall cost of simulation tools.
Shared Simulation Centers	Establish centralized training hubs where multiple institutions can collaborate and share access to high-fidelity simulation equipment, optimizing resource use.
Cloud-Based AI Platforms	Integrate AI-powered virtual training environments hosted in the cloud, allowing trainees to engage in remote, flexible practice sessions without on-site hardware.

By increasing the affordability and accessibility of simulation tools through these measures, vascular trainees, regardless of their location or institutional resources, can benefit from high-quality surgical education. This inclusive approach is essential for ensuring global competency in AA surgery and maintaining consistent training standards across the field [52,53].

Long-Term Impact of Simulation on AA Surgical Outcomes

Despite the increasing adoption of simulation-based training in vascular surgery, there remains a limited amount of long-term data assessing its direct impact on real-world surgical outcomes. While numerous studies have shown that simulation can enhance technical proficiency and boost confidence among trainees, it is not yet fully established whether these improvements consistently translate into better patient outcomes, lower complication rates, or reduced operative times, particularly in complex procedures such as AA repair [52].

To address this knowledge gap, several key areas require further investigation. One important research priority is to assess patient outcomes among surgeons who received simulation-based training compared to those who followed traditional apprenticeship models. This would provide more conclusive evidence on the clinical effectiveness of simulation. Another critical focus should be on measuring the retention of surgical competencies over time, evaluating whether skills developed through simulation are maintained in the long term. Additionally, determining the optimal frequency and timing of simulation sessions is essential to ensure they effectively supplement real-world surgical experience without becoming redundant or underutilized.

As vascular surgery education continues to evolve, adopting a data-driven approach to evaluating simulation-based training will be vital. Robust research in this area can help refine educational frameworks, justify institutional investments in advanced simulation technologies, and, most importantly, contribute to improving patient safety and surgical care outcomes across the board.

AR in AA Training

Augmented reality (AR) overlays are another emerging tool that enhances real-time visualization during live surgeries. AR-based systems can superimpose 3D anatomical reconstructions over fluoroscopic images, providing surgeons with enhanced spatial awareness and procedural accuracy. This technology is particularly valuable in complex aortic cases, such as fenestrated and branched EVAR, where precise endograft positioning is crucial to avoid organ ischemia or device failure. AR together with AI can also be integrated into simulation-based learning platforms, where AI can track a trainee’s hand movements, response time, and decision-making skills to provide objective competency assessments [54].

Evaluating the Impact of Simulation on Surgical Outcomes

A growing body of evidence supports the positive impact of simulation-based training on surgical performance and patient outcomes. Studies have demonstrated that trainees who undergo simulation training make fewer procedural errors, require less fluoroscopy time, and achieve better technical precision compared to those trained solely through traditional methods. In the context of EVAR, simulation training has been shown to streamline procedural workflows, improve stent-graft deployment accuracy, and reduce radiation exposure, benefiting both surgeons and patients [13,17,18].

Despite these promising findings, the long-term impact of simulation on AA surgical outcomes remains an area for further research. Large-scale, multi-center studies are needed to validate the long-term benefits of simulation-based training on perioperative mortality, complication rates, and reintervention rates. Additionally, the guidelines call for the development of standardized simulation-based competency assessments, ensuring that trainees meet consistent skill thresholds before performing high-risk AA repairs [15,36]. *Limitations*

This review has several limitations that should be acknowledged. First, as a narrative review, it relies on the synthesis of existing literature without performing a systematic search or meta-analysis. This approach may introduce selection bias and limit the ability to draw quantitative conclusions about the efficacy of simulation-based training in AA surgery. Second, the inclusion of diverse simulation modalities, ranging from VR and computational modeling to robotic-assisted platforms, may dilute the focus on specific training methods and their comparative effectiveness. Third, while the review highlights promising innovations such as AI-driven platforms and patient-specific modeling, the evidence supporting their widespread integration into vascular surgery curricula remains limited and often based on small-scale or single-center studies. Additionally, the review does not address the variability in training environments and resource availability across different geographic and institutional settings, which may affect the generalizability of its recommendations.

## Conclusions

Simulation-based training has emerged as a cornerstone of modern AA surgery education, bridging the gap between declining operative exposure and rising procedural complexity. By integrating VR, AI, 3D printing, and robotic systems, current platforms offer high-fidelity environments that enhance technical skills, clinical decision-making, and procedural readiness. While challenges remain in standardization, cost, and access, ongoing research and technological innovation continue to expand simulation’s role in vascular surgery. As personalized modeling and real-time feedback become routine, simulation is poised to elevate surgical training, optimize patient outcomes, and define the next generation of competency-based vascular education.
